# 3D-Hepatocyte Culture Applied to Parasitology: Immune Activation of Canine Hepatic Spheroids Exposed to *Leishmania infantum*

**DOI:** 10.3390/biomedicines8120628

**Published:** 2020-12-18

**Authors:** Armanda V. Rodrigues, Graça Alexandre-Pires, Ana Valério-Bolas, David Santos-Mateus, Mariana Rafael-Fernandes, Maria A. Pereira, Dário Ligeiro, Telmo Nunes, Raquel Alves-Azevedo, Marcos Santos, Isabel Pereira da Fonseca, Gabriela Santos-Gomes

**Affiliations:** 1 Global Health and Tropical Medicine, GHTM, Instituto de Higiene e Medicina Tropical, IHMT, Universidade Nova de Lisboa, UNL, Rua da Junqueira 100, 1349-008 Lisboa, Portugal; armanda.rodrigues@ihmt.unl.pt (A.V.R.); ana.bolas@ihmt.unl.pt (A.V.-B.); david.s.mateus@gmail.com (D.S.-M.); marianarfernandes90@gmail.com (M.R.-F.); mapereira@esav.ipv.pt (M.A.P.); alves.raquel.ra@gmail.com (R.A.-A.); 2 CIISA—Centro de Investigação Interdisciplinar em Sanidade Animal, Faculdade de Medicina Veterinária, Universidade de Lisboa, Avenida da Universidade Técnica, 1300-477 Lisboa, Portugal; gpires@fmv.ulisboa.pt (G.A.-P.); socramitis@msn.com (M.S.); ifonseca@fmv.ulisboa.pt (I.P.d.F.); 3 Agrarian School, Polytechnic Institute of Viseu, Quinta da Alagoa-Estrada de Nelas Ranhados, 3500-606 Viseu, Portugal; 4 IPST-Centro de Sangue e Transplantação de Lisboa, Alameda das Linhas de Torres 117, 1749-005 Lisbon, Portugal; dario@ipst.min-saude.pt; 5 Microscopy Center, Faculty of Sciences, University of Lisbon, Campo Grande, 1749-016 Lisboa, Portugal; telmonunes@hotmail.com

**Keywords:** 3D cell culture, hepatocytes, immune response, *Leishmania infantum*, meglumine antimoniate

## Abstract

The application of innovative three-dimensional (3D) spheroids cell culture strategy to Parasitology offers the opportunity to closely explore host–parasite interactions. Here we present a first report on the application of 3D hepatic spheroids to unravel the immune response of canine hepatocytes exposed to *Leishmania infantum*. The liver, usually considered a major metabolic organ, also performs several important immunological functions and constitutes a target organ for *L. infantum* infection, the etiological agent of canine leishmaniasis (CanL), and a parasitic disease of major veterinary and public health concern. 3D hepatic spheroids were able to sense and immunologically react to *L. infantum* parasites, generating an innate immune response by increasing nitric oxide (NO) production and enhancing toll-like receptor (TLR) 2 and interleukin-10 gene expression. The immune response orchestrated by canine hepatocytes also lead to the impairment of several cytochrome P450 (CYP450) with possible implications for liver natural xenobiotic metabolization capacity. The application of meglumine antimoniate (MgA) increased the inflammatory response of 3D hepatic spheroids by inducing the expression of Nucleotide oligomerization domain (NOD) -like receptors 1 and NOD2 and TLR2, TLR4, and TLR9 and enhancing gene expression of tumour necrosis factor α. It is therefore suggested that hepatocytes are key effector cells and can activate and orchestrate the immune response to *L. infantum* parasites.

## 1. Introduction

In living tissue, cells exist in three dimensional (3D) microenvironments, with intricate cell-to-cell and cell-to-matrix interactions, creating a dynamic and complex network of nutrient transport, waste removal, and cell signaling pathways. The mimicking of this microenvironment has long been a major concern in research as standard two dimensional (2D) or monolayer cell cultures may inadequately represent cells’ complex interrelations, which make these culture systems less reliable predictors of in vivo cell behavior [1]. Spheroids are a type of 3D cell modeling that mimic the in vivo cell’s microenvironmental conditions, and overcome some insufficiencies seen in 2D monolayer cultures. Culturing cells as 3D spheroids can induce in cells the establishment of natural gradients of oxygen, nutrients, metabolites, and soluble signals, mirroring the in vivo tissue, thus enabling the differentiation of heterogeneous cell populations. Spheroids also have homogenous geometry and optimal physiological cell-to-cell and cell-to-extracellular matrix interactions [2]. The application of this culture system to hepatocytes, which are very complex cells that rapidly dedifferentiate in vitro conditions, has shown improved results compared to the traditional 2D culture methods [3,4]. Therefore, 3D spheroids constitute a closer resemblance to in vivo tissue in terms of cellular communication and the development of extracellular matrices are a valuable tool to study organ complexity.

The application of innovative cell culture methods has been mainly driven by the pharmaceutical industry and its need to test new drugs for toxicity [2,5]. The translation of this innovation to parasitological studies has been slower and here is presented as a first report on the application of 3D spheroids to unravel the immune response of canine hepatocytes exposed to *Leishmania infantum*.

*L. infantum* is the aetiological agent of human zoonotic visceral leishmaniasis (ZVL) and canine leishmaniasis (CanL), a parasitic disease of major public health concern since both ZVL and CanL can be fatal if left without treatment. Naturally infected dogs, which constitute the main parasite reservoir, may remain asymptomatic for long periods before clinical signs develop. But once the disease becomes patent, progression is usually rapid, and death occurs within a few weeks to months. Clinically affected dogs usually exhibit characteristic clinical features such as skin lesions, loss of weight, lymphadenopathy, anemia, and/or renal failure [6]. One of the main target organs in CanL is the liver. The liver is largely constituted by hepatocytes, which are highly active metabolic cells that play critical roles in synthesizing molecules essentials to support body homeostasis, regulate energy balances, detoxify the body from endo or exogenous molecules, in addition to the role played as active immune cells. Hepatocytes play a key role in controlling systemic innate immunity via production and secretion of acute-phase proteins (APPs) and complement components found in plasma, likewise by the expression of several pattern recognition receptors (PRRs). These proteins can either directly kill pathogens or orchestrate the immune system for efficient pathogen clearance [7,8]. During the acute phase or the systemic inflammatory response, a variety of pro-inflammatory cytokines, such as interleukin (IL)-6, IL-1β, IL-22, tumor necrosis factor (TNF)-α, and interferon (IFN)-γ can stimulate hepatocytes to produce high levels of complement, secreted APPs, and express PRRs [9]. Expression of genes encoding these proteins is controlled by liver-specific transcription factors, such as hepatocyte nuclear factor (HNF) −1 and 4 and CCAAT enhancer-binding protein [10,11]. Toll-like receptors (TLRs) have been detected on all types of liver cells, particularly in hepatocytes, which are recognized to express messenger RNAs for all known TLRs. TLRs are likely involved in the uptake and clearance of endotoxins, the production of pro- and anti-inflammatory cytokines, and generation of reactive oxidative stress [12,13]. These innate immune receptors are transmembrane protein complexes with domains leucine-rich that are activated by recognition of pathogen-associated molecular patterns (PAMPs). Following dimerization of the TLR, these domains attract the adapter protein MyD88 or MD-2, activating a cascade signal leading to translocation of the transcription factors NF-κB or AP-1 to the cell nucleus, directing the expression of pro-inflammatory cytokines [14,15]. Several other cytoplasmic PRRs have been identified in hepatocytes, including Nucleotide oligomerization domain (NOD) -like receptors and the retinoic acid-inducible gene 1 (RIG)-like helicases [12]. However, the role of these PRRs in hepatocytes in the context of CanL has not yet been fully understood.

Cytochromes P450 (CYP450) constitute a superfamily of heme enzymes found in the lysosomal compartment of cells in several organs. The phase I detoxification system, composed mainly by the cytochrome P450 supergene family, are usually, the first enzymatic defense against foreign compounds. Most drugs are metabolized through phase I biotransformation, however, phase II metabolization enzymes also play an important role in the biotransformation of endogenous compounds and xenobiotics into more easily excreted forms, as well as in the metabolic inactivation of pharmacologically active substances. Drug metabolism via the cytochrome P450 system has emerged as an important determinant in the occurrence of several drug interactions that can result in increased drug toxicity, reduced pharmacological effect, or adverse drug reactions [16,17,18,19]. Recent attention has been drawn to the effect of the immune response on the regulation of CYP450 metabolization capacity [20]. In the present study, a first approach to how the immunological response generated in the liver can influence the metabolization of meglumine antimoniate (MgA), a leishmanicidal drug widely used for CanL treatment, was evaluated. MgA is a classical anti-leishmanial drug belonging to the pentavalent antimonial class. Although the use of this drug has potential serious hepatic side effects, the MgA mechanism of action is still not fully understood. Currently, the most accepted model indicates that MgA promotes oxidative stress, culminating in glutathione (GSH) depletion and damage of parasite DNA [21]. Immune modulatory properties are also described for MgA such as the induction of inflammatory immune response [22,23]. However, at the hepatic level, uncontrolled or excessive inflammation can lead to liver damage and can even be life-threatening.

Therefore, the present work constitutes a step forward from the previous study [24], as it takes advantage of the innovative 3D cell culture technique and applies it to canine hepatocytes, aiming to unravel the immune role played by dog hepatocytes when exposed to *L. infantum* parasites and the impact of inflammation on the metabolic activity of these cells.

## 2. Methods

### 2.1. Hepatocyte Isolation

Canine (*Canis lupus familiaris*, *n* = 10) hepatocytes were isolated by a two-step perfusion protocol followed by collagenase digestion and Percoll^®^ gradient centrifugation, as previously described [3,4,24]. Briefly, a liver lobule was cleaned of internal blood by perfusion, and tissue digestion was performed with collagenase H (Roche Diagnostics, Mannheim, Germany). The cellular suspension was filtered through sterile gauze and centrifuged, and pellets were resuspended in Williams’E medium (Sigma-Aldrich, Saint Louis, MO, USA). Cells (3.5 × 10^6^ cells.mL^−1^) were overlaid into 20 mL of a solution of 25% Percoll^®^ (GE Healthcare Bio-Sciences AB, Uppsala, Sweden) in 1× phosphate-buffered saline (PBS) solution and centrifuged. Viable hepatocytes were resuspended in supplement Williams’E medium [Penicillin (100 U/mL)/streptomycin (100 µg.mL^−1^) (Sigma-Aldrich), 1.4 µM hydrocortisone (Sigma-Aldrich), 15 mM HEPES (Sigma-Aldrich), 1 mM sodium pyruvate (Sigma-Aldrich), 1 mM non-essential amino acids (NEAA) (Biochrom GmbH, Irvine, United Kingdom), 40 µg.mL^−1^ gentamycin (Sigma-Aldrich), and 10% (*v*/*v*) fetal bovine serum (FBS, Sigma-Aldrich)]. This procedure allowed the isolation of a solution of pure hepatocytes with ≈80% viability.

The dogs included in the present study were collected from a natural population with no restrictions on breed, gender, or age to reflect the natural diversity of the canine population.

### 2.2. Leishmania Infantum Parasites

*L. infantum* zymodeme MON-1 (MHOM/PT/89/IMT151) were cultured in Schneider drosophila (SCHN) medium with L-glutamine (Sigma-Aldrich) supplemented with 10% (*v*/*v*) of heat-inactivated FBS, and penicillin-streptomycin (Sigma-Aldrich) at 100 U/mL and 100 μg.mL^−1^ respectively (complete SCHN medium) at 24 °C. Only virulent parasites with less than five passages were used [25].

### 2.3. D-Hepatocyte Culture and Exposure to L. infantum

A 3D-hepatic spheroids culture was established in a 250 mL Spinner vessel (Wheaton) following hepatocyte isolation. Cells (1.2 × 10^5^ cells.mL^−1^) were inoculated in supplemented Williams’E medium extra supplemented with 15% of FBS (*v*/*v*) to promote cell aggregation. At 24 h post-inoculation, half of the medium volume was replaced with fresh medium and at 72 h the total medium of the vessel was replaced with a fresh medium. The cell suspension was collected in 50 mL tubes and centrifuged at 50× *g* for 10 min at room temperature. Pelleted cells were resuspended in fresh culture medium and inoculated again in the spinner vessel to ensure no loss of cells. The spinner was maintained at 50 rpm rotation in a cell incubator (MCO-18AIC, Sanyo, Osaka, Japan) at 37 °C in a humidified atmosphere with 5% CO_2_.

Hepatocytes seeded in a 24 well-plate at 5 × 10^5^ cell.mL^−1^ (2D system) with fresh medium replaced at 24, 48, and 72 h post-isolation were used as control of the 3D system. After 72 h in culture, 3D- and 2D-cultured hepatocytes were exposed to *L. infantum* parasites in a proportion of 3:1 promastigotes to hepatocytes. Parasites were left to interact with hepatocyte for 72 h. Samples of medium and cells were collected at 1.5, 3, 5, 24, 48, and 72 h of incubation and used for the determination of several parameters. Cells were followed by optical microscopy observation under an inverted microscope (Olympus, CKX41, Olympus Corporation, Tokyo, Japan), and digital images were acquired using an Olympus CS30 camera.

### 2.4. Meglumine Antimoniate

Meglumine antimoniate (MgA) is commercially available as Glucantime^®^ (Merial, France), in a formulation constituted by 81 mg of the compound/mL. After 72 h in contact with *L. infantum* virulent promastigotes, the medium of 3D-hepatocyte culture was removed, and the cell aggregates were resuspended in supplemented William’s E fresh medium with 100 µM of MgA. Cells were left to incubate for more than 72 h at 37 °C in a humidified atmosphere with 5% CO_2_. Samples of medium and cells were collected at 1.5, 3, 5, 24, 48, and 72 h of incubation for the evaluation of several parameters.

### 2.5. Immunostaining of Spheroids

Suspension of hepatic spheroids (1 mL) was collected and centrifuged at 50× *g* for 10 min. Spheroids were washed with cold 1× PBS, fixed with 2% paraformaldehyde (*m*/*v*) in PBS for 20 min on ice, and then cells were washed with cold PBS. Cell spheroid permeabilization was performed overnight at 4 °C with a solution of PBS 1% Tween-20 (*v*/*v*) (Sigma-Aldrich) and 0.2% fish gelatin (*m*/*v*) (Sigma-Aldrich). For antibodies incubation, a solution of PBS 0.125% fish gelatin (*m*/*v*) and 1% Tween-20 (*v*/*v*) was used. The incubation with primary antibodies was performed for 90 min at room temperature, kept in the dark. For observation of cell morphology, actin goat polyclonal antibody Fluorescein isothiocyanate (FITC) conjugated (sc-1616, Santa Cruz Biotechnology, CA, USA), raised against a peptide at the C-terminus of actin of human origin was used in a dilution of 1:500 according to the manufacturer’s recommendation. Cells were washed three times with PBS 0.08% tween-20 (*v*/*v*) and finished with three more washes with 1× PBS. For ferritin detection, a goat polyclonal antibody (sc-14416, Santa Cruz Biotechnology, CA, USA) raised against a peptide near the N-terminus of the ferritin-heavy chain of human origin was used in a 1:100 dilution according to the manufacturer’s instructions. The polyclonal anti-actin and anti-ferritin antibodies were described as having cross-reactivity with several species, including the dog. For the anti-ferritin staining, a secondary rabbit polyclonal anti-goat IgG Alexa Fluor^®^ 647 (ab150143, Abcam, Life Technologies Corporation, CA, USA) antibody was used at a dilution of 1:500. Between the primary and secondary antibodies, cells were washed three times with buffer PBS 0.08% tween-20 (*v*/*v*) followed by three more washes in 1× PBS. Control slides were prepared with only one antibody staining. Slides were observed under a Leica TCS SP2 laser scanning confocal microscope, and digital images were acquired.

### 2.6. Scanning Electron Microscopy 

Hepatocyte spheroids suspension (1 mL) was collected and washed with cold 1× PBS and fixed with 2% paraformaldehyde (*m*/*v*) (Sigma-Aldrich) in PBS for 20 min on ice. Cells were then washed with cold 1× PBS by centrifugation at 350× *g* for 10 min. Spheroids were filtrated through a Millipore mesh and subsequently dehydrated in a graded ethanol (Merck KGa, Darmstadt, Germany) series [30, 50, 70, 80, 90, and 100% (*v*/*v*)]. Samples were dried using the critical point drying method, coated with gold–palladium, and mounted on stubs. Cells were then observed in a scanning electron microscope (JEOL5200-LV, JSM Electron Microscopes, Tokyo, Japan), and digital images were acquired.

### 2.7. CYP450 Activity Assay

The activity of alkoxyresorufin O-dealkylation (AROD) of the CYP450 family (phase I enzymes), 7-ethoxyresorufin (EROD) (canine CYP1A1 CYP1A2), 7-methoxyresorufin (MROD) (canine CYP1A2 CYP1B), 7-pentoxyresorufin (PROD) (canine CYP2B11), and 7 benzyloxyresorufin (BROD) (canine CYP2B11 CYP3A12 CYP3A26), as well as uridine 5′-diphospho-glucuronosyltransferase (UDP-glucuronosyltransferase, UGT) (phase II), were analysed. EROD, MROD, PROD, BROD, and UGT reactions were performed at all the sampling time points of this study (non-exposed 2D and 3D culture system, *L. infantum* exposed, and drug-treated 3D-hepatocyte spheroids). All substrates (resorufin-methyl-ether, resorufin-penthyl-ether, resorufin-ethyl-ether, and resorufin-benzyl-ether) dissolved in DMSO (10 µM) were from Sigma-Aldrich. 4-methylumbelliferone (4-MU) (Sigma-Aldrich) was dissolved in William’s E culture medium (10 mM). For each reaction, 1 mL of cell suspension was sampled from the spinner vessel and 10 µl of the correct substrate was added. Samples were collected in duplicate. Cell suspensions were then incubated for 1 h at 37 °C, allowing substrate metabolization by hepatocytes. After incubation, samples were centrifuged at 500× *g* for 10 min at 4 °C and supernatants were collected and stored at −20 °C until further analysis. After thawed, the samples were vigorously vortexed and 200 µL of each sample were plated in duplicate in a sterile 96 well black plate (Nunc™, Thermo Scientific™, Waltham, MA, USA). A standard curve with resorufin (Sigma-Aldrich), the product of the O-dealkylase reaction, was established in William’s E medium. Sample fluorescence was analysed in triplicate on a fluorometer ((TRIADTM 1065, DYNEX technologies, Chantilly, VA, USA) with excitation and emission wavelengths of 535 nm and 595 nm, respectively. For the UGT assay, a standard curve with 4-methylumbelliferone (4-MU) was performed, and 4-MU mobilization was calculated by subtracting the amount detected in samples to the initial quantity of 4-MU. A blank prepared with William’s E medium and an assay control made with each substrate was added to the fresh culture medium (free of hepatocytes) and incubated for 1 h at 37 °C. Samples were analysed in triplicate in a fluorometer (BIOTEK® FLx800, Vermont, WI, USA) with excitation at 360/40 nm and emission at 460/40 nm.

### 2.8. Urea Detection

Urea concentration was analysed in the supernatant of cell samples with the colorimetric commercial kit QuantiChrom™ Urea Assay Kit-DIUR-500 (BioAssay System, Hayward, CA, USA) according to the manufacturer’s instructions. The 3D-hepatocyte suspension (1 mL) was collected in duplicate and centrifuged to clean off the cells. Supernatants were stored at −20 °C until further analysis. After thawed, samples were vigorously vortexed and 50 µL of each sample was plated in triplicate in a sterile 96 well transparent plate (Nunc™, Thermo Scientific™, Waltham, MA, USA) For posterior analysis, the sterile and supplemented William’s E culture medium absorbance was subtracted from each sample reading.

### 2.9. Nitrite Detection

The commercial kit Nitrate/Nitrite Colorimetric Assay kit (Abnova, Taipei, Taiwan) was used according to the manufacture’s recommendation to quantify the nitrate/nitrite concentration in cell supernatants. Samples of hepatocyte in 2D and 3D culture were taken in duplicate and centrifuged to clean cells from the medium. Supernatants were collected and stored at −20 °C until further analysis. The blank performed with sterile and supplemented William’s E culture medium also was included in the plate and the production of nitric oxide (NO) was estimated following the manufacturer’s instructions.

### 2.10. Real-Time PCR Analysis

Gene expression of innate immune receptors (NOD1, NOD2, TLR2, TLR4, and TLR9) and cytokines (IL-10, IL-6, and TNF-α) were quantified by real-time polymerase chain reaction (PCR) quantitative method. The primers and protocols used were described by Rodrigues and co-workers [24]. Briefly, RNA extraction was performed using the NZY Total RNA Isolation Kit (Nzytech genes & enzymes, Lisboa, Portugal) and processed into cDNA synthesis, using NZY First-strand cDNA Synthesis Kit (Nzytech genes & enzymes) following the manufacturer’s indications. Real-time PCR was performed in a 7500 FAST Real-Time PCR System thermal cycler (Applied Biosystems, Foster City, CA, USA). Amplification was carried out in a total volume of 20 µL, containing 2 µL of cDNA, 10 µL of SensiFAST SYBR Lo-ROX (Bioline Reagents Ltd, Meridian Bioscience, London, United Kingdom), and primers (20 pmol.µL^−1^). External cDNA standards were constructed for all target genes by cloning PCR fragments, generated by the same primers, into a pGEM^®^-TEasy Vector according to the manufacturer’s recommendations (Promega, Madison, WI, USA) and as described by Rodrigues et al. [26]. The number of copies of each gene and sample was normalized to the housekeeping gene β-actin to regularize differences in relative quantities of initial cDNA in the sample. Results were expressed as the log of the number of gene copies per 1000 copies of β-actin.

### 2.11. Statistical Analysis

To explore the differences between time-points and experimental conditions the non-parametric Wilcoxon test was used for two related samples. All the data analysis was performed using the software GraphPad Prism 7, taking into consideration a significance level of 5% (*p* < 0.05). Throughout the present study, several outliers were registered as a reflex of the dogs’ natural population.

### 2.12. Ethical Considerations

The present study was carried out using dead animals. Liver samples were obtained from a local shelter, from euthanized dogs due to aggressiveness and lack of adoption. As no live animals were used, there was no purpose to consult the Commission on Ethics and Animal Wellbeing for advice for this particular study.

## 3. Results

### 3.1. In Vitro Generation of Canine Hepatic Spheroids

**Figure 1:**
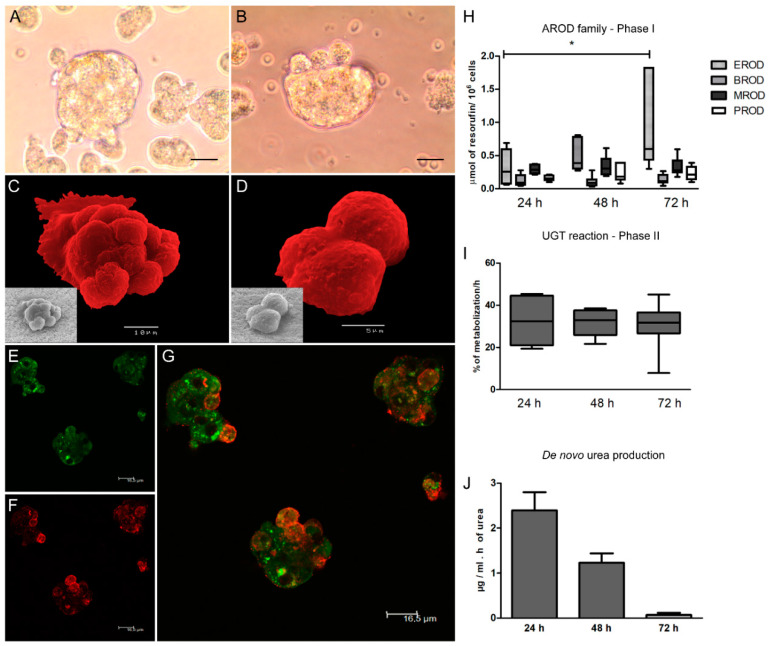
Morphological and metabolic characterization of the hepatic spheroids. Samples of hepatocytes cultured in a 3D-system for 72 h were observed under an inverted microscope (**A**,**B**), (scale bar 20 µm, 400× magnification) and scanning electron microscope (**C**,**D**) (scale bar 10 µm and 5 µm, respectively). (**C**,**D**) Images were artificially colored to evidence cell morphology. Cellular aggregates exhibit a smooth membrane bound by an extracellular matrix generated by cells. Spheroids stained for actin (green) (**E**) and ferritin (red) (**F**) were observed under a fluorescent confocal microscope, and images were acquired. (**G**) represents the overlapping of actin (**E**) and ferritin (**F**) staining (Scale bar 16.5 µm for E, F and G images). Hepatic spheroids with diverse levels of complexity and nearly complete cell fusion can be observed. Nuclei—black spots; actin network—green; ferritin—red. The enzymatic activity of 7-ethoxyresorufin (EROD), 7 benzyloxyresorufin (BROD), 7-methoxyresorufin (MROD), 7-pentoxyresorufin (PROD) (**H**) of the phase I alkoxyresorufin O-dealkylation (AROD) family, and UGT (**I**) of phase II represented by box plots, whiskers (minimum to maximum), and urea de novo production (**J**), expressed by the mean and standard error, were evaluated at 24, 48, and 72 h of 3D-hepatocyte culture. Results of 10 dogs and three replicates per sample are represented. The non-parametric Wilcoxon matched-pairs signed-rank test was applied for statistical comparisons, and * indicates statistical significance values (*p* < 0.05).

Taking advantage of the spinner vessel mechanics in conjugation with the natural aggregation properties evidenced by hepatocytes, it was possible to generate hepatic spheroids within 72 h post-isolation (Figure 1A,B). Cellular spheroids were observed, constituted by several cells, ranging in size between 30 and 50 µm, and showing smooth membranes bound by an extracellular matrix generated by cells that constitute the compact hepatic aggregates (Figure 1C,D).

Hepatic aggregates stained for actin and ferritin (Figure 1E–G) revealed a three-dimensional cell arrangement, with cell superposition and possible fusion of cell membranes. Actin staining highlighted the cytoplasmic network feature with some dense dots, probably due to secretory vesicles. Ferritin spots were also identified in the spheroids, indicating possible ferritin storage vesicles.

The activity of alkoxyresorufin O-dealkylation (AROD) of the CYP450 family (phase I enzymes), uridine 5′-diphospho-glucuronosyltransferase (UDP-glucuronosyltransferase, UGT) (phase II enzyme), and urea production were analysed, as they constitute characteristics of viable and differentiated hepatocytes. The 3D-cell system allowed hepatocytes to recover rapidly from isolation trauma and acquire their natural CYP450 enzyme activity. After 24 h of culture, EROD (CYP1A1 and CYP1A2) activity showed a time-dependent increase (P_72 h_ = 0.0418). BROD (CYP2B11, CYP3A12, and CYP3A26), MROD (CYP1A2 and CYP1B), and PROD (CYP2B11) exhibited stable activity (Figure 1H). Phase II enzymes assessed by a UGT reaction evidenced steady and similar metabolization levels throughout the 72 h of incubation after hepatocyte isolation (Figure 1I). 2D-cultured hepatocytes needed extra time to recover (48 to 72 h in culture), achieving a similar EROD activity level compared to the 3D-culture system (Supplementary Figure S1). Regarding urea production, a metabolic characteristic of hepatocytes, 3D spheroids exhibited urea production (Figure 1J). However, as the medium of 3D-hepatocytes only was replaced after the first 72 h, urea accumulation occurred, leading to a relative decrease in the urea *de novo* production rate.

Altogether, these results evidenced that in vitro 3D conditions established in the present study allowed dog hepatocytes to constitute cellular spheroids with viable and metabolically active cells, allowing the continuation of the study with the evaluation of immune parameters.

### 3.2. L. infantum Parasites Perturb the Regular Metabolic Function of Hepatic Spheroids

**Figure 2:**
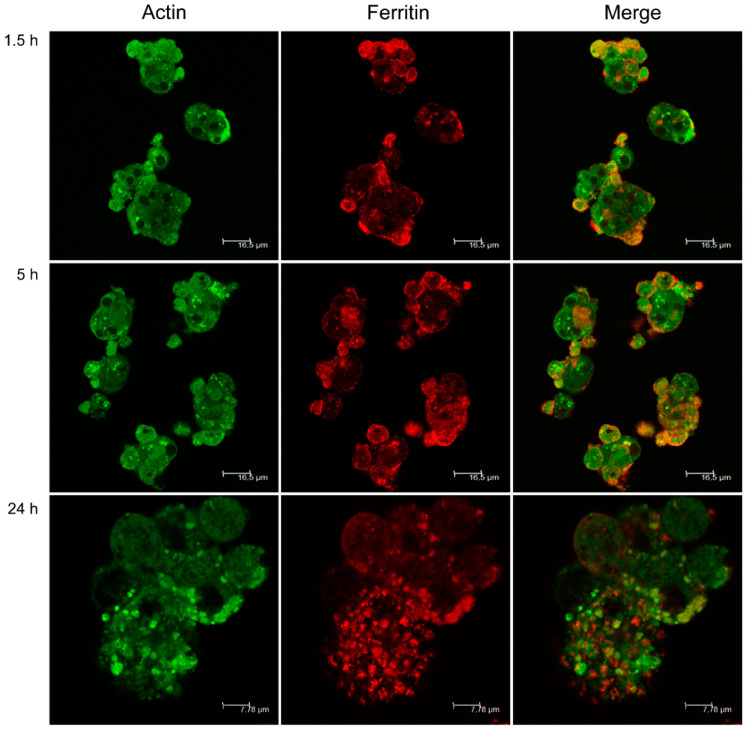
Actin and ferritin of hepatocyte aggregates exposed to *L. infantum* promastigotes. 3D-culture samples obtained at 1.5, 5, and 24 h of parasite exposure were stained with actin antibody conjugated with FITC and anti-ferritin antibody was conjugated with Alexa Fluor 647. Aggregates were observed under a confocal fluorescent microscope, and images were acquired. The density of actin vesicles (green) increased after 24 h of exposure to the parasite, and ferritin (red) deposits increased after 5 h of parasite exposure followed by a decrease after 24 h of exposure to promastigotes. Black spots—hepatocyte nucleus.

**Figure 3:**
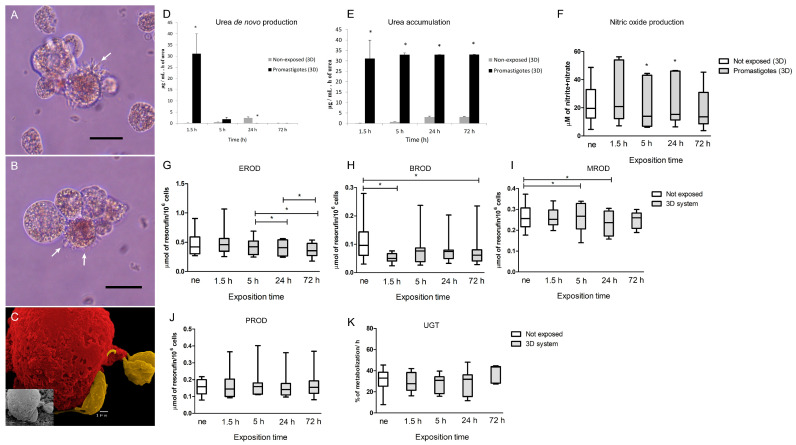
3D-hepatic spheroids exposed to *L. infantum* promastigotes. 3D-culture samples obtained at 5 h (**A**) and 24 h (**B**,**C**) of parasite exposure were observed under an inverted optical microscope (scale bar 20 µm, 400× magnification) and by scanning microscopy. Parasites were found in suspension in the medium and in close contact with the hepatic spheroids (white arrows) (**A**,**B**), which can be observed in more detail in (**C**) (Scale bar 1µm). Artificial color was applied on C to cells (red) and *L. infantum* parasites (yellow). De novo production of urea (**D**) by aggregates and urea accumulation (**E**) at 1.5, 5, 24, and 72 h of promastigote exposure and by spheroids non-exposed to parasites are represented by the mean and standard error. NO production by hepatic spheroids at 1.5, 5, 24, and 72 h of *L. infantum* exposure and by aggregates non-exposed to parasites (**F**) is represented by box plots, and whiskers (minimum to maximum). Phase I alkoxyresorufin O-dealkylation (AROD) activity was evaluated by EROD (**G**), BROD (**H**), MROD (**I**), and PROD (**J**) assays in hepatic spheroids exposed to *L. infantum* and non-exposed (ne) to parasites is represented by box plots and whiskers (minimum to maximum). Phase II UGT (**K**) enzyme activity was evaluated at 24, 48, and 72 h of parasite exposure. Data are represented by box plots and whiskers (minimum to maximum). Samples of 10 dogs were performed in triplicate, and the non-parametric Wilcoxon test was used for statistical comparisons (*p* < 0.05). The symbol * indicates statistical significance values when comparing non-exposed spheroids vs. promastigote-exposed spheroids.

After the establishment of viable and functional 3D-hepatic spheroids, cells were exposed to *L. infantum* promastigotes, and biochemical and immune parameters were evaluated. It was observed that *L. infantum* promastigotes established a close interaction with hepatocytes and remained strongly attached to the cell membrane (Figure 2 and Figure 3A–C).

Cellular spheroids exposed to *L. infantum* promastigotes were stained for actin and ferritin (Figure 2). Actin revealed the cytoskeleton organization of cellular spheroids, highlighting cell superposition and strong cohesion of the spheroid. In conjugation with the time of parasite exposure, an increase in hepatocyte vesiculation (green dots) was observed, particularly visible after 24 h of exposition. Ferritin spots (in red) were observed in spheroids exposed to promastigotes. After 24 h, ferritin appeared to be stored in vesicles located in hepatocyte cell borders, probably as a result of increased capture of iron from the culture medium.

The interaction of *L. infantum* promastigotes with the hepatic spheroids led to an early transitory burst of urea production (*p* = 0.0156) (Figure 3D). At 24 h of parasite exposure, the *de novo* urea production was apparently abolished when compared with non-exposed spheroids (*p* = 0.0156). During the exposition to *L. infantum*, urea accumulation was highly increased (*p* = 0.0156) (Figure 3E) compared with non-exposed spheroids.

As an important immune signaling molecule, nitric oxide was also assessed. Hepatic spheroids non-exposed to inflammatory stimuli produced residual NO, however, when exposed to promastigotes for 5 h and 24 h (*p* = 0.0313) significantly higher levels of NO were detected (Figure 3F). The 3D spheroid system enables hepatocytes to activate the production of NO in higher levels than those observed in 2D cultures (Supplementary Figure S2). These findings indicate that the close cell-to-cell contact of *L. infantum* parasites with the hepatic spheroids may have implications in the immune activation of the cells and oxidative stress. Hepatic spheroids showed a tendency to reduce CYP450s enzymatic activity, when in the presence of *L. infantum* promastigotes. EROD (CYP1A1 and CYP1A2) revealed a progressive decrease in enzyme activity (*p* = 0.0319) when exposed to *L. infantum* parasites (Figure 3G). BROD assay (CYP2B11, CYP3A12, and CYP3A26) in spheroids exposed to *L. infantum* also revealed early suppression of enzymatic activity (P_1.5 h_ = 0.0156; P_72 h_ = 0.0391), observed since the early time of interaction with the parasite (Figure 3H). MROD (CYP1A2 and CYP1B) activity registered a significant decrease after 5 h of promastigote exposure (*p* = 0.0469) followed by another significant decrease at 24 h (*p* = 0.0469) (Figure 3I). PROD (CYP2B11) assay did not exhibit important changes (Figure 3J). Phase II enzyme (UGT) showed a stable metabolization rate compared to non-exposed spheroids (Figure 3K).

Thus, *L. infantum* promastigotes impact regular hepatocyte activity, disturbing urea production, CYP450s metabolism as well as NO production and ferritin storage.

### 3.3. Canine Hepatic Spheroids Activate the Innate Immune Response against L. infantum Parasites

**Figure 4:**
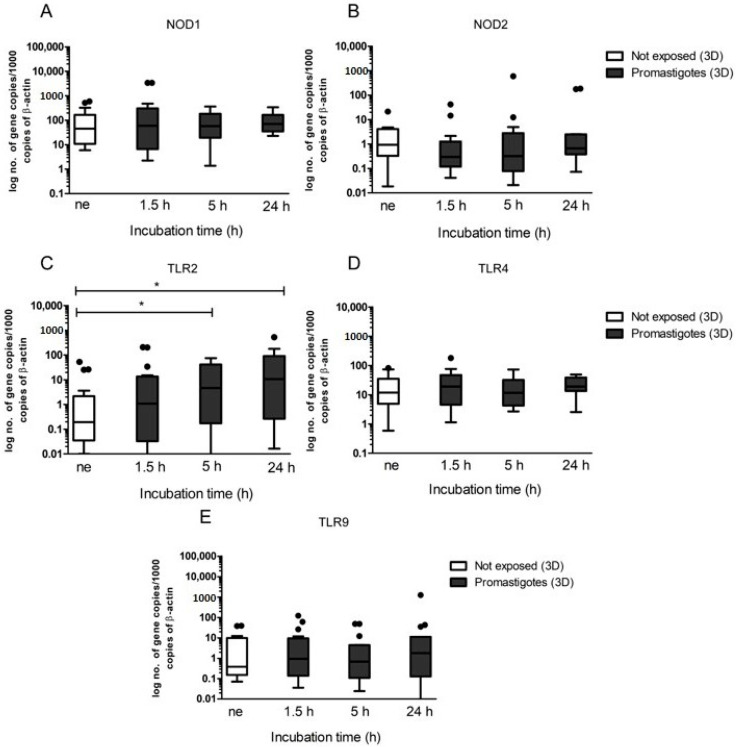
Gene expression of innate immune receptors by canine hepatic spheroids exposed to *L. infantum* virulent promastigotes. Samples of the 3D-cultured hepatocytes were used to evaluate mRNA accumulation of NOD1 (**A**), NOD2 (**B**), TLR2 (**C**), TLR4 (**D**), and TLR9 (**E**) at 1.5, 5, and 24 h of exposure to promastigotes. In parallel, *L. infantum* non-exposed spheroids (ne) were also evaluated. Results of the 10 dogs performed in triplicate are represented by Tukey graphs. Black dots are indicative of outlier values. The non-parametric Wilcoxon test was used for statistical comparisons, and * indicates statistical significance values (*p* < 0.05).

Immune activation of hepatic spheroids by contact with *L. infantum* promastigotes was assessed by real-time PCR. NOD1 (Figure 4A) and NOD2 (Figure 4B) gene expression did not differ from spheroids not exposed to promastigotes throughout the incubation time. However, when compared to hepatic spheroids not exposed to virulent promastigotes, TLR2 gene expression registered a significant increase in promastigote-exposed spheroids for 5 h (*p* = 0.0105) and 24 h (*p* = 0.0353), indicating that exposition to the parasite triggers the TLR2 receptor (Figure 4C). On the contrary, the amounts of TLR4 and TLR9 mRNA did not reveal major changes in spheroids exposed to parasites, during the entire observation period (Figure 4D,E). Taking together, these results indicate that dog hepatocytes can recognize *L. infantum* antigens through TLR2, where other TLRs and NODs, evaluated in the present study may not be activated by parasite PAMPs.

**Figure 5:**
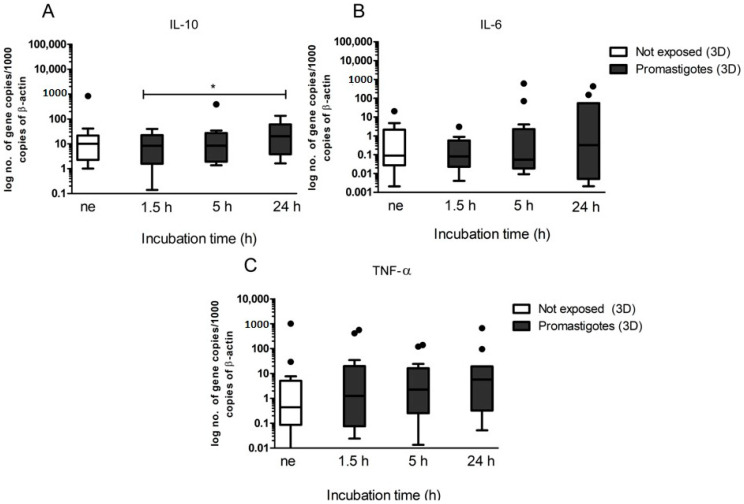
Gene expression of IL-10, IL-6, and TNF-α by canine hepatic spheroids exposed to *L. infantum* virulent promastigotes. Samples of 3D-cultured hepatocytes were used to evaluate the mRNA accumulation of IL-10 (**A**), IL-6 (**B**), and TNF-α (**C**) at 1.5, 5, and 24 h of exposure to promastigotes. In parallel, aggregates not-exposed (ne) to parasites also were evaluated. Results of 10 dogs performed in triplicate are represented by Tukey graphs. Black dots are indicative of outlier values. The non-parametric Wilcoxon test was used for statistical comparisons, and * indicates statistically significant values (*p* < 0.05).

Gene expression of the anti-inflammatory cytokines, IL-10 (Figure 5A), IL-6 (Figure 5B), and of pro-inflammatory TNF-α (Figure 5C) were assessed in 3D hepatic spheroids exposed to *L. infantum* promastigotes. In comparison with spheroids not-exposed to promastigotes, *L. infantum*-exposed hepatic spheroids did not reveal important changes during the entire observation period. IL-6 and TNF-α cytokines tended to increase with parasite exposition time, but only was detected a significant increase in IL-10 gene expression (*p* = 0.0161) after 24 h of promastigotes exposure. These results may be related to superimposition and aggregation of hepatic spheroids, which limit parasite interaction with the outer membrane of the spheroid and favour the immune tolerant microenvironment that is naturally promoted by hepatocytes.

### 3.4. Dog Hepatic Spheroids Become Activated by Parasite Antigens and Nucleic Acids Released during MgA Treatment

Hepatic spheroids cultured in a 3D-system and exposed to *L. infantum* promastigotes for 72 h were treated with MgA to evaluate the impact of a leishmanicidal drug in hepatocyte spheroids, as this constitutes a good liver model.

**Figure 6:**
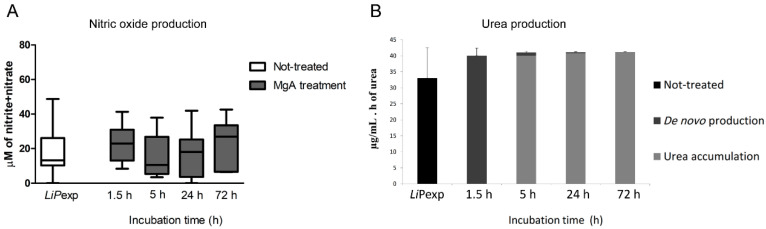
Nitric oxide and urea production by *L. infantum* exposed spheroids treated with meglumine antimoniate (MgA). Hepatic spheroids exposed to *L. infantum* promastigotes for 72 h were treated with MgA. At different time points, samples of culture medium were analysed for nitric oxide (NO) (**A**) and urea production (**B**). Untreated hepatocyte aggregates exposed to parasites for 72 h (*Li*Pexp) were also used to evaluate NO. Results of samples of 10 dogs performed in triplicate are represented by box plots, whiskers (minimum to maximum), and median in the case of NO, and by the mean and standard error in the case of urea.

The addition of the drug did not impact the morphology of the hepatic spheroids, as cells remained attached by the extracellular matrix. Urea production by promastigote-exposed spheroids followed the anterior described pattern (Figure 1J), producing an initial burst when facing a stress situation (Figure 6B). Furthermore, MgA treatment did not cause significant changes in *de novo* synthesis urea. Likewise, NO production in the 3D-culture system was not altered by the addition of MgA, presenting identical high production levels during the 72 h of treatment (Figure 6A). These levels were similar to NO production, observed in not treated promastigote-exposed spheroids.

**Figure 7:**
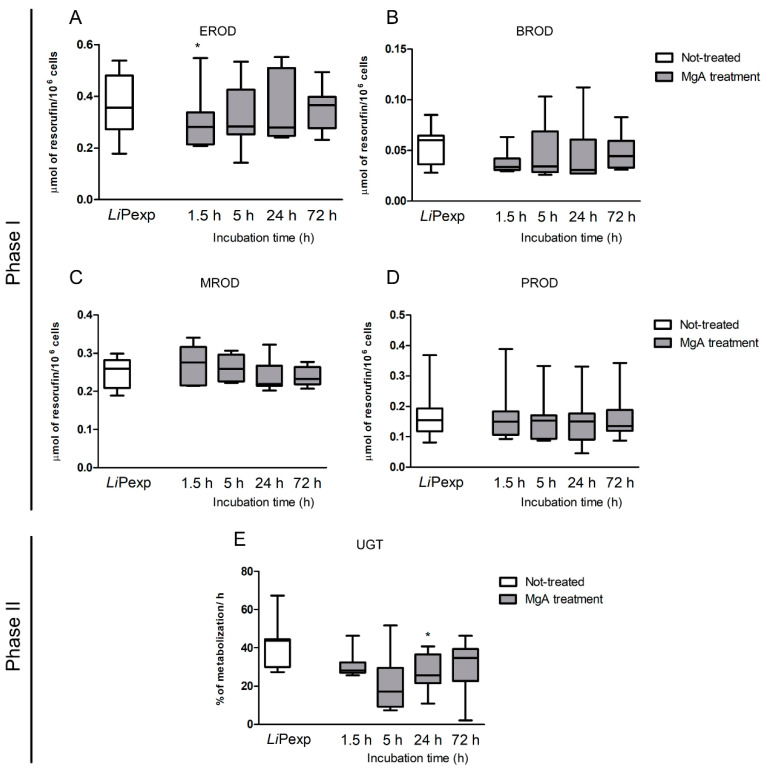
Phase I and phase II enzyme activity after MgA treatment of hepatic spheroids exposed to *L. infantum* axenic promastigotes. Samples of 3D-culture supernatants were used to evaluate EROD (**A**), BROD (**B**), MROD (**C**), PROD (**D**), and UGT (**E**) enzyme activity at 1.5, 5, 24, and 72 h of MgA treatment. Untreated hepatocyte aggregates exposed to parasites for 72 h (*Li*Pexp) also were evaluated. Results of 10 dogs performed in triplicate are represented by box plots, whiskers (minimum to maximum), and medium. The non-parametric Wilcoxon test was used for statistical comparisons, and * indicates statistically significant values (*p* < 0.05) when comparing untreated vs. treated amastigote-exposed spheroids.

Phase I enzyme of promastigote-exposed spheroids treated with MgA exhibited activity levels similar to untreated promastigote-exposed spheroids (Figure 7), except for EROD metabolization that evidenced a significant decrease (*p* = 0.0391) at the beginning of treatment (1.5 h) in comparison with untreated hepatocytes (Figure 7A). Phase II enzymes exhibited a decreasing tendency with statistical significance after 24 h incubation with MgA (*p* = 0.0234) (Figure 7E).

**Figure 8:**
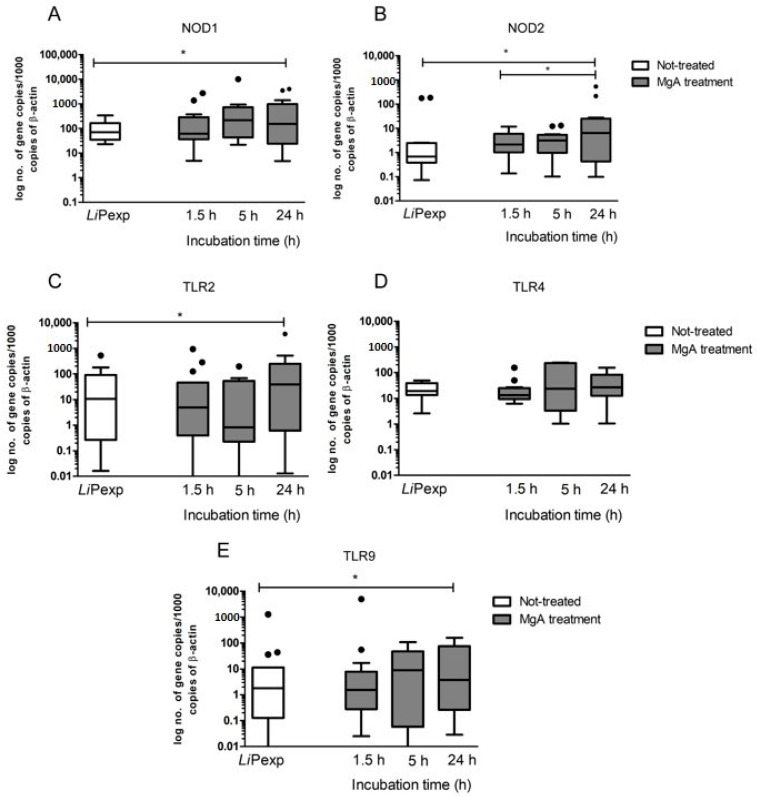
Gene Expression of innate immune receptors after MgA treatment of promastigote-exposed spheroids. Samples of 3D-hepatocytes treated with MgA were collected to evaluate NOD1 (**A**), NOD2 (**B**), TLR2 (**C**), TLR4 (**D**), and TLR9 (**E**) mRNA accumulation at 1.5, 5, and 24 h of treatment. Untreated hepatocyte aggregates exposed to parasites for 72 h (*Li*Pexp) also were evaluated. Results of 10 dogs performed in triplicate are represented by Tukey graphs. Black dots are indicative of outlier values. The non-parametric Wilcoxon test was used for statistical comparisons, and * indicates statistically significant differences (*p* < 0.05).

Gene expression of the innate immune receptors was assessed after the addition of MgA to hepatic spheroids previously exposed for 72 h to *L. infantum* promastigotes. When compared with parasite-exposed and untreated spheroids, the addition of MgA induces a significant increase of NOD1 gene expression (*p* = 0.0342) after 24 h incubation with the drug (Figure 8A). NOD2 exhibited a similar tendency with increased mRNA accumulation after 24 h of MgA treatment (*p* = 0.0166) when compared with not-treated *L. infantum* exposed spheroids and spheroids treated for 1.5 h (*p* = 0.0049) (Figure 8B). The addition of the leishmanicidal drug also increases TLR2 gene expression that reaches significant values at 24 h of treatment (*p* = 0.0353) (Figure 8C). On the other hand, TLR4 expression levels did not reveal differences caused by the addition of MgA. Nevertheless, TLR9 gene expression registered an increase after 24 h of MgA treatment when compared with untreated spheroids (*p* = 0.0353) (Figure 8E). These results suggest that NOD receptors and TLRs, with exception of TLR4, may play a key role in recognizing parasite antigens.

**Figure 9:**
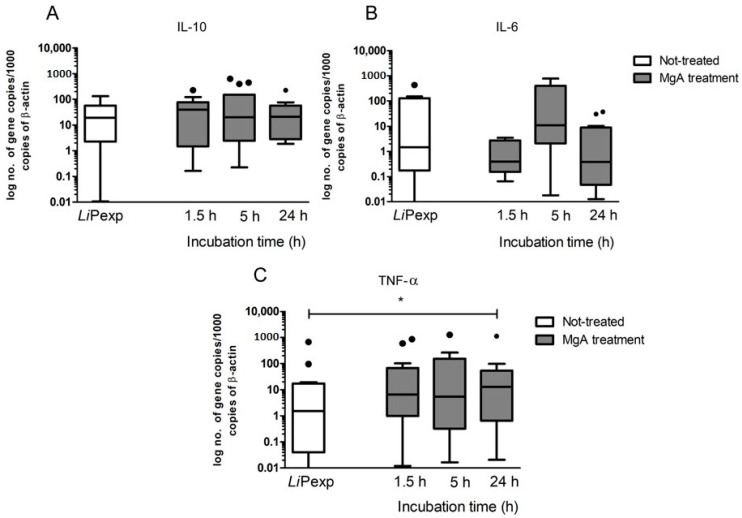
Gene expression of IL-10, IL-6, and TNF-α after MgA treatment of hepatic spheroids exposed to *L. infantum* virulent promastigotes. Samples of promastigote-exposed spheroids treated with MgA were used to evaluate mRNA accumulation of IL-10, (**A**), IL-6 (**B**), and TNF-α (**C**) at 1.5, 5, and 24 h after treatment. Untreated hepatocyte aggregates exposed to parasites for 72 h (*Li*Pexp) also were evaluated. Results of 10 dogs performed in triplicate are represented by Tukey graphs. Black dots are indicative of outlier values. The non-parametric Wilcoxon test was used for statistical comparisons, and * represents statistically significant values (*p* < 0.05).

The increase of innate immune receptors gene expression was followed by changes in the profile of cytokine gene expression. After MgA treatment of parasite-exposed spheroids, gene expression of the anti-inflammatory IL-10 and IL-6 (Figure 9A,B) were similar to the untreated promastigote-exposed spheroids. However, IL-6 exhibited a transient increase at 5 h incubation and a high gene expression of pro-inflammatory TNF-α (*p* = 0.0031) was registered after 24 h of treatment (Figure 9C).

Altogether, these findings demonstrated that early treatment with MgA is capable of inducing changes in the gene expression of innate receptors and cytokine profile of hepatic spheroids previously exposed to *L. infantum* parasites.

## 4. Discussion

In recent years 3D-cell culture has gradually become a prominent method for cellular studies mainly due to its ability to mimic the in vivo microenvironment of cells in tissues. The generation of 3D-multicellular spheroids is, therefore, an attempt to mimic the in vivo liver architecture, enhancing cell-to-cell and cell-to-matrix interactions, recapitulating many in vivo tissue structures and functions. Hepatocytes rapidly recover from isolation trauma in the 3D system, as indicated by CYPs activity early after hepatocyte isolation (24 h), urea production, and the presence of internal vesicles of ferritin, demonstrating that hepatic spheroids were constituted by differentiated and active hepatocytes. In the present work, an innovative approach to parasite-tissue interaction was used, by applying, for the first time, 3D cell culture methodology to generate canine hepatic spheroids, allowing obtaining a detailed and reliable characterization of the interaction between *L. infantum* and canine hepatocytes.

In our study, special emphasis was applied to the immune response generated by canine hepatocytes to *L. infantum*. Although not considered a major immunological organ, the liver has been recently acknowledged to house several immune functions and even to be able to orchestrate an immune response [27]. The liver is a main target organ in ZVL and CanL, but in contrast to the spleen, that stays chronically infected and allows parasite replication, infection in the liver is normally self-containing in asymptomatic individuals. *Leishmania* infected liver´s usually presents several well-organized granulomas, walling off the parasites, exhibiting an effective immunity in an environment of effector T cells. Sanchez and co-workers [28] showed that livers of asymptomatic and naturally *L. chagasi* (syn *L. infantum*) infected dogs exhibited a predominance of Th1 CD4^+^ and CD8^+^ T cells, CD45RO^+^CD44^low^ (central memory) T cells and CD45^+^CD44^+^ effector T cells, together with dendritic cells, expression of MHC class II, and CD11c and CD18 integrins. This cellular microenvironment is characterized by high IFN-γ, IL-12, and TNF-α production through CD4^+^ T cells [29,30]. By contrast, in symptomatic animals liver granulomas are described to be non-organized and to have a cellular infiltrate composed of T cells and other heavily parasitized cells that are non-effective [28,31]. However, the role of hepatocytes to control the canine infection by *Leishmania* is yet poorly studied. Hepatic resistance in ZVL and CanL is also attributed to the generation of reactive nitrogen (NO) and oxygen intermediates (ROS), potent leishmanicidal molecules, both of which have been shown to play a role in containing parasite growth during the early stages of infection [31,32]. Our results indicate that hepatocytes were able to recognize the presence of the pathogen by increasing NO production, PRRs, and cytokine expression, orchestrating an immune response. The inducible nitric oxide synthase (iNOS) is an enzyme isoform that may generate large quantities of NO and can be induced in a variety of cell types, including hepatocytes, by inflammatory stimuli, such as TNF-α, IL-1, and IL-6 [33,34]. *L. infantum* is known to have several mechanisms to subvert the host immunological response, in particular, this parasite exhibits the ability to control NO production since it is a potent leishmanicidal molecule. This parasite usually stimulates the production of urea, as frequently seen in blood macrophages and in Kupffer cells [22,35], subverting the host immune system to promote a Th2 response,. In the current study, canine hepatic spheroids increased NO production in the presence of *L. infantum*, which was accompanied by the tendency to increase the expression of pro-inflammatory cytokines TNF-α and IL-6, indicating an immune recognition and activation of these cells towards the parasite. These findings are similar to what was previously observed by our group [24]. Interestingly, there are differences in the magnitude of the immune response to *L. infantum* generated by canine hepatic spheroids (3D) and traditional 2D cultured hepatocytes used in the previous study. In this case, the immune reaction of 2D canine hepatocytes was more exacerbate than the 3D-aggregates. Both studies strongly indicated that hepatocytes can recognize the presence of the pathogen and react by orchestrating an inflammatory immune response. However, the liver is characterized by an environment of immunological tolerance, mainly due to its frequent exposure to antigens from the intestinal tract. Since the liver constitutes a major metabolic organ, impairment of liver functions may have a higher impact on individual health. Therefore, to avoid excessive damage by inflammation and its drastic consequences to the organ, hepatocytes usually promote immune tolerance [36,37]. In the current study, we observed a significant increase in anti-inflammatory IL-10 that possibly can balance the inflammatory response and avoid cellular damage. It seems that hepatic spheroids mimic the natural tolerance of the liver to inflammatory stimulus (presence of *L. infantum*), probably due to increased cell-to-cell and cell-to-matrix contact, which enables a more in vivo like hepatocyte behavior.

Hepatocytes express several membrane-bound PPRs, such as extracellular TLR2 and TLR4, intracellular TLR9, and cytoplasmatic NODs. TLRs and NODs can be triggered by exogenous ligands derived from pathogens or by endogenous ligands resulting from cellular injury, activating innate immunity. Their activation is often associated with the production of pro-inflammatory cytokines and the generation of effector molecules, which promote differentiation of Th1 cells, leading to inflammatory responses. As a result, transcription of certain pro-inflammatory immune mediators including IL-6, IL-12, IL-23, and TNF-α are induced [38]. TLRs and NODs are well established essential components of the innate immune system and facilitate the early detection of many infections. However, the role played by these receptors in CanL remains not fully understood, as studies describing TLR and NODs transcription during infection are still scarce. Therefore, the present study may constitute a breakthrough by contributing to the understanding of their role in canine hepatocyte immune response to *L. infantum*. Several studies have addressed the role of TLRs in different tissues of naturally and experimentally infected dogs. Montserrat-Sangrà and colleagues described the up-regulation of TLR2 transcription in blood, suggesting the activation of the innate and pro-inflammatory immune response [39]. TLR9 and FoxP3 were up-regulated in dog skin at the early stages of infection and in lymph nodes and significant downregulation of TLR3, TLR4, TLR9, IL-17, IL-22, and FoxP3 transcription was found associated with disease progression [40]. Recently, increased frequency and expression of TLR9 was associated with a lower parasite load in the jejunum of *L. infantum* infected dogs, whereas the colon showed a higher parasite load along with an increased frequency and expression of TLR2 [41,42]. Fewer studies have addressed the role of TLRs in the dog liver during *L. infantum* infection. Hosein et al. [40] describe increased TLR2 transcription and TLR9 downregulation in the hole liver tissue of experimentally infected dogs. Furthermore, our findings indicate that *L. infantum* parasites can establish a close interaction with hepatic spheroids, upregulating TLR2, confirming that this innate immune receptor has a non-negligible role in *Leishmania* infection, at least in the early stages of infection and suggesting activation of a pro-inflammatory response. Our findings also indicate that the close contact of *L. infantum* parasites with hepatic spheroids may have implications not only in the metabolism and immune activation of hepatocytes but also gives support to the hypothesis of parasite internalization by hepatocytes, as suggested by previous works [24,43]. However, the present study used promastigotes, the vector-transmitted *L. infantum* form, more commonly found to interact with macrophages, neutrophils, and other white blood cells. In natural circumstances, hepatocytes would hardly directly interact with promastigotes, but with disseminated amastigotes from lysed parasitized cells. This fact may constitute a technical limitation of the presented work. A previous work performed by our group [24], addressed the innate immune response of canine hepatocyte exposed to *L. infantum* axenic amastigotes and virulent promastigotes in vitro, using the 2D system. The authors reported that both parasite forms were able to activate the innate immune response of canine hepatocytes, apparently using different innate immune receptors. Interestingly, the present study also observed that promastigotes in 3D culture conditions start to exhibit morphological changes and differentiate in amastigote-like forms. Although not yet completely proved, but with growing experimental evidence [24,43,44,45], the internalization of *L. infantum* by canine hepatocytes may redefine the role of hepatocytes in CanL and consequently question the importance of the liver, as a possible reservoir organ for the parasite, mainly because of its immunotolerant microenvironment favoring parasite survival.

Apart from the immunological functions, the liver is mainly recognized as a major metabolic organ and CYP450s constitute the major drug-metabolizing enzymes. These enzymes are responsible for the metabolization of endogenous molecules such as hormones and exogenous and potentially harmful compounds. The immunological state has been recently added as a possible influencer factor of CYPs active state [20]. In most inflammatory conditions, CYP450s activities are suppressed, but some are unaffected, and others can be induced under inflammatory conditions. As some drugs must be converted into their pharmacological or toxicological active metabolites by CYP450 enzymes, suppression of their metabolism during an inflammatory response can lead to a reduced therapeutic or toxic effect [46,47]. The findings of the present study revealed that the presence of *L. infantum* tends to upregulate gene expression of the pro-inflammatory cytokines IL-6 and TNF-α by canine hepatocytes, directing hepatic CYPs to a pro-inflammatory immune environment, and therefore, decreasing their activity. However, the inflammation is also balanced by the expression of IL-10, which promotes tolerance to the parasite and may avoid accentuated decrease of CYPs activity, remaining similar to control (non-exposed hepatocytes). Inhibition of CYPs by inflammation was particularly evident in the case of EROD (CYP1A1 and CYP1A2), BROD (CYP2B11, CYP3A12, and CYP3A26), and MROD (CYP1A2 and CYP1B) assays. As dogs constitute the peridomestic reservoir of *L. infantum*, understanding how the major metabolic enzymes of the liver react in the presence of this parasite, might be useful to predict drug metabolization profiles during treatment. Our results suggest that CYP1A1 and CYP1A2 (measured in EROD assay), highly induced by the administration of MgA are probably involved in the metabolization of MgA or of a metabolite derivate from it CYP2B11, CYP3A12, and CYP3A26 (measured in BROD assay) also tended to increase, showing a possible role in MgA metabolization. Understanding how canine hepatocytes are influenced by the inflammatory response can help to better direct the treatment. 

Antileishmanial treatment impacts hepatocyte immune response, affecting cytokine generation and expression of innate immune receptors. MgA treatment increases NOD1, NOD2, TLR2, and TLR9 gene expression and generates TNF-α, which coincides with drug leishmanicidal activity. As NOD1, NOD2, and TLR9 are intracellular innate immune receptors, these findings suggest that parasite killing by MgA ensures the exposure of hepatocytes to other parasite antigens, which are recognized by innate sensors, affecting the immune response by increasing the inflammatory component.

Although the 3D-hepatocyte system does not represent the entire complexity of the liver or the organism, it constitutes an effective tool contributing to understanding how hepatocytes react and orchestrate the immune response to *L. infantum* parasites. Our findings confirm that hepatocytes are active cells in innate immune surveillance and can initiate an immune response, being therefore key players in the liver’s early response to *L. infantum* infection and contributing to parasite control. Altogether, these findings add new knowledge for a better understanding of the liver as an innate immunological key organ.

## Supplementary Materials

The following are available online at https://www.mdpi.com/2227-9059/8/12/628/s1, Figure S1: Phase I and phase II enzyme activity of canine hepatocytes, after isolation, cultured in 2D and 3D systems. Figure S2: Nitric oxide production by canine hepatocytes exposed to *L. infantum* in 2D and 3D systems.
